# Neurovascular Impairment in Type 2 Diabetes Mellitus: The Role of Adipocyte-Derived Exosomes

**DOI:** 10.3390/biom16020233

**Published:** 2026-02-03

**Authors:** Harshal Sawant, Ji Chen Bihl

**Affiliations:** Department of Biomedical Sciences, Joan C. Edwards School of Medicine, Marshall University, Huntington, WV 25755, USA

**Keywords:** type 2 diabetes mellitus, adipocyte-derived exosomes, cerebrovascular complications, stroke susceptibility, cognitive impairment, neurovascular dysfunction, oxidative stress, mitochondrial dysfunction

## Abstract

Type 2 diabetes mellitus (T2DM) is a major metabolic disorder characterized by chronic hyperglycemia with far-reaching morbidities. Among these, diabetes-related cerebrovascular complications such as ischemic and hemorrhagic stroke, cerebral blood vessel disease, and vascular dementia are significant contributors to morbidity and mortality. Adipose tissue is a metabolically active endocrine organ that becomes dysfunctional in T2DM and communicates with distant tissues via secreted factors, including extracellular vesicles such as exosomes (EXs), phospholipid bilayer-enclosed nanosized particles. These adipocyte-derived exosomes (Ad-EXs) carry bioactive cargo, including lipids, proteins, and microRNAs that influence the function of distant organs, including the brain. Evidence indicates that Ad-EXs in T2DM are a significant risk factor for cerebrovascular complications via neurovascular impairment either directly through the adipose tissue–brain axis or indirectly by other organs. This review provides an overview of current knowledge on how Ad-EXs from different adipocyte populations contribute to cerebrovascular complications through oxidative stress, blood–brain barrier disruption, neuroinflammation, and mitochondrial dysfunction. Particular emphasis is placed on recent findings and gaps in knowledge linking diabetic Ad-EXs with brain microvascular endothelial cells that mediate neurovascular crosstalk, contributing to stroke susceptibility and cognitive decline. We also discuss the potential of Ad-EXs as biomarkers and therapeutic targets for cerebrovascular complications of T2DM.

## 1. Introduction

Type 2 diabetes mellitus (T2DM) is a progressive metabolic disorder characterized by chronic hyperglycemia resulting from a combination of insulin resistance and impaired insulin secretion due to pancreatic β-cell dysfunction. It is considered a global burden with over 95% of T2DM cases of diabetes worldwide and has reached epidemic proportions due to the rising prevalence of obesity, sedentary lifestyles, and unhealthy dietary patterns. According to the World Health Organization, approximately 828 million people are suffering from T2DM [1], and 3.4 million deaths are attributed to it in the year 2024 [2]. Persistent hyperglycemia, along with associated metabolic abnormalities such as dyslipidemia, oxidative stress, and chronic low-grade inflammation, leads to widespread vascular and organ damage in T2DM.

**Figure 1:**
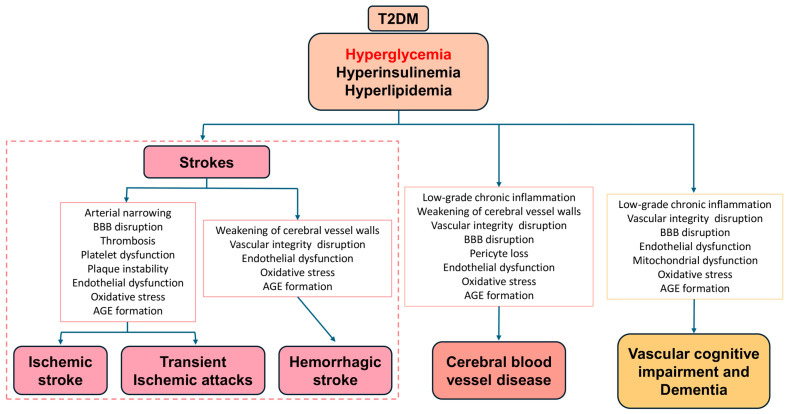
Cerebrovascular complications of T2DM. T2DM is often signified as a hyperglycemic condition leading to various cerebrovascular complications such as ischemic stroke, transient ischemic attack, hemorrhagic stroke, cerebral blood vessel disease, vascular cognitive impairment, and dementia. Abbreviations—T2DM: Type 2 diabetes mellitus; BBB: blood–brain barrier; AGE: advanced glycated end product.

Complications of T2DM are broadly categorized into microvascular and macrovascular disorders [3]. Microvascular complications include damage to small blood vessels leading to diabetic retinopathy (progressive loss of vision), nephropathy (impaired kidney function), and neuropathy (impaired nerve conduction). Macrovascular complications include damage to large vessels leading to atherosclerosis, coronary artery disease, peripheral arterial disease, and cerebrovascular diseases, all of which markedly increase cardiovascular morbidity and mortality. In addition, T2DM is associated with non-vascular complications such as increased susceptibility to infections, impaired wound healing, and certain cancers. Among all complications of T2DM, cerebrovascular disease represents a major cause of morbidity and mortality in diabetic populations [4]. Chronic hyperglycemia, dyslipidemia, oxidative stress, low-grade inflammation, and endothelial dysfunction synergistically promote the formation of advanced glycated end products (AGE), atherogenesis, and impaired cerebrovascular autoregulation. These pathophysiological changes substantially increase the risk of ischemic stroke [5], hemorrhagic stroke [6], transient ischemic attacks [7], cerebral blood vessel disease [8], vascular cognitive impairment, and dementia [9] (Figure 1). Furthermore, T2DM often coexists with other cardiovascular risk factors such as hypertension and obesity, amplifying the risk and severity of cerebrovascular events.

Among the organs involved in the pathophysiology of T2DM, adipose tissue plays a central role not only as the largest energy storage unit but also as an active endocrine organ. Adipose tissue is classified as white, beige, or brown based on morphological characteristics. Further classification of white adipose tissue (WAT) is made according to anatomical location, with the two major depots being subcutaneous WAT, situated beneath the skin, and visceral (or omental) WAT, which resides within the abdominal cavity surrounding internal organs. Adipose tissue is predominantly made up of adipocytes along with extracellular matrix and stromal vascular fraction, which is made up of macrophages, vascular endothelial cells, and fibroblasts. The adipose tissue dysfunction in T2DM is marked by hypertrophy and hyperplasia of adipocytes, hypoxia, increased infiltration of immune cells, and a shift toward an altered or pro-inflammatory secretome, including extracellular vesicles [10,11]. Extracellular vesicles (EVs) are membrane-bound, lipid bilayer nanoparticles secreted by all eukaryotic cells into the extracellular environment, creating a cell-free path for inter-organ communication. They carry bioactive cargo such as nucleic acids, proteins, and lipids [12]. In T2DM, adipocyte dysregulation leads to aberrant secretion of adipokines (e.g., increased leptin, resistin, and TNF-α, and decreased adiponectin) and circulating free fatty acids. These alterations impair insulin signaling in peripheral organs, including the liver, muscle, and pancreatic tissues, thereby exacerbating systemic insulin resistance, a hallmark of T2DM. Beyond classical metabolic target organs, emerging evidence suggests that adipose tissue-derived extracellular vesicles, including adipocyte-derived exosomes (Ad-EXs), act as critical mediators linking dysfunctional adipose tissue to multi-organ (including vascular and neurovascular) injury [13,14,15]. Specifically, dysfunctional adipose tissue causes oxidative stress, endothelial dysfunction, and ectopic fat deposition in non-adipose organs such as the liver, heart, and brain via circulating Ad-EXs [16]. Thus, targeting adipose tissue dysfunction, particularly adipocyte dysfunction and their released Ad-EXs, represents a promising strategy for improving insulin sensitivity, preventing complications, and reducing the overall burden of T2DM. In this review, we summarized the current state of knowledge with gaps in understanding the role of Ad-EXs in cerebrovascular complications of T2DM.

## 2. Exosomes in Intercellular Communications

### 2.1. General Biology of Exosomes

The International Society of Extracellular Vesicles (ISEV), via its ‘minimal information for studies of extracellular vesicles’ (MISEV 2023) guidelines [17], has defined ‘extracellular vesicles’ (EVs) as lipid bilayer nanoparticles released by all cells (both prokaryotes and eukaryotes) without the ability to self-replicate. According to MISEV2023, EVs should not be classified solely based on size into exosomes, microvesicles, or apoptotic bodies, as vesicle biogenesis cannot be reliably inferred from size alone. Instead, the guidelines recommend the use of operational terminology, classifying EVs as small EVs (sEVs; <200 nm) or medium/large EVs (m/lEVs; ≥200 nm), unless their intracellular origin or mechanism of release is experimentally demonstrated. Among these EVs, exosomes (EXs) originate from the endosomal pathway, specifically through the inward budding of multivesicular bodies (MVBs), which subsequently fuse with the plasma membrane of the cell to release exosomes into the extracellular space. Unlike apoptotic bodies or microvesicles, exosomes are formed through a regulated, energy-dependent process, enabling selective packaging of bioactive molecules. The molecular cargo of exosomes includes proteins (tetraspanins, heat shock proteins, Major Histocompatibility Complex molecules), lipids (cholesterol, sphingomyelin, ceramides), and nucleic acids (mRNAs, microRNAs, long non-coding RNAs, and DNA fragments) [18]. This diverse cargo reflects the physiological state of the parent cell and enables exosomes to serve as messengers in intercellular communication. Exosomes are taken up by recipient cells through mechanisms such as receptor–ligand interactions, direct membrane fusion, or endocytosis. Once internalized, their cargo can modulate signaling pathways, gene expression, and cellular functions. Through these processes, exosomes play crucial roles in maintaining tissue homeostasis, regulating immune responses, and coordinating metabolic and neurovascular functions. Dysregulation of exosome biogenesis, cargo composition, or signaling is implicated in the pathogenesis of various diseases, including cancer, neurodegeneration, and metabolic disorders such as T2DM [19]. Exosomes are increasingly being recognized for their potential as both a biomarker and therapeutic agent in T2DM and associated complications.

### 2.2. Adipocyte-Derived Exosomes

Adipocyte-derived exosomes (Ad-EX) are a specialized subset of extracellular vesicles secreted by adipocytes of adipose tissue, an organ known as a primary energy reservoir of the body and now recognized as a key regulator of systemic metabolic homeostasis. Adipocytes originate from preadipocytes via the process of adipogenesis (or differentiation), which is found to alter the exosomal contents depending on the differentiation stage [20]. A study by Connolly et al. showed that murine 3T3-L1 preadipocytes have high levels of preadipocyte factor-1 (PREF1) and peroxisome proliferator-activated receptor gamma (PPARγ) at preadipogenesis and, upon cell differentiation, show the highest levels of adiponectin at day 15 [21]. They also reported that Ad-EX production is higher before adipogenesis of 3T3-L1 cells. The exosomes released from the adipocytes encapsulate and transport a variety of bioactive molecules—including adipokines, lipids, proteins, mRNAs, and non-coding RNAs (particularly microRNAs; miRNAs)—that reflect the physiological or pathological state of the adipose tissue. Durcin et al. reported a distinct characteristic of Ad-EXs from 3T3-L1 cells, where, with a proteomics study, they identified 168 proteins, and with a lipidomics study, they identified total lipid content as 58% phosphatidylcholine (PC), 7% phosphatidylethanolamine (PE), 4% phosphatidylinositol (PI), 2% phosphatidylserine (PS), 1.775% sphingolipids [including 0.35% ceramides (Cer), 0.025% glycosylceramides (GlyCer), and 1.4% diacylglycerols (DGs)], and 22.9% sphingomyelin (SM) [22]. Apart from the proteins and lipids, Ad-EXs are considered an excellent source of miRNAs. In fact, Thomou et al. reported that adipose tissue is a major source of exosomal miRNAs in circulation by performing serum analysis of wild-type and adipose tissue-specific knockout of miRNA-processing enzyme Dicer (ADicerKO) [23]. Ad-EXs function as potent mediators of intercellular communication between adipose tissue and distant organs such as the liver, skeletal muscle, pancreas, vasculature, and brain. Through the transfer of their cargo, they modulate insulin signaling, glucose and lipid metabolism, inflammatory responses, and vascular function.

In conditions such as obesity and T2DM, the secretion profile and molecular content of Ad-EX become dysregulated. They often exhibit pro-inflammatory and pro-atherogenic properties, contributing to systemic insulin resistance, endothelial dysfunction, and increased risk of cardiovascular and cerebrovascular complications [24]. Thus, Ad-EXs represent both a critical link in adipose tissue–organ communication and a promising source of biomarkers and therapeutic targets in metabolic and vascular diseases.

### 2.3. Types of Adipocytes and Their Derived Exosomes

Adipocytes, being the most predominant component of adipose tissue, have a major role in its unique morphological, metabolic, and endocrine functions. The diversity of adipocytes not only determines energy balance and thermogenesis but also shapes the composition and biological activity of Ad-EXs. Significant functional, morphological, and metabolic heterogeneity exists between adipocytes from different depots, such as white, brown, and beige fat. These variations are influenced by their distinct developmental origins, genetic factors, hormonal regulation, and microenvironment. Even within white adipose tissue (WAT), adipocytes from different anatomical depots exhibit distinct properties. For example, visceral adipose tissue (VAT) is more metabolically active and lipolytic in nature than subcutaneous adipose tissue (SAT) in metabolic conditions like obesity and T2DM [25]. Interestingly, Fang et al. reported an association of T2DM with an increase in the fraction of small adipocytes with a corresponding decrease in large adipocytes in both SAT and VAT in humans [26]. A clinical study conducted by Hu et al. demonstrated evidence that an increase in the VAT to SAT ratio is a risk factor for carotid atherosclerosis, a cardiovascular disease in T2DM patients [27]. Considering these various pieces of evidence, studying Ad-EXs from different adipose deposits would aid in unraveling the mechanistic heterogeneous roles of different adipose deposits in T2DM. The major adipocyte types include the following:

#### 2.3.1. White Adipocytes

White adipocytes are unilocular cells with large lipid droplets, specialized in storing excess energy as triglycerides and secreting adipokines such as adiponectin, resistin, and leptin. They secrete extracellular vesicles, including exosomes enriched with lipids, adipokines, and microRNAs that influence insulin sensitivity, inflammation, and systemic metabolism in obesity and T2DM [28]. The canonical markers of white Ad-EXs are endosomal sorting proteins (ALIX, TSG101), tetraspanins (CD9, CD63, CD81), and EV-associated heat shock proteins (HSP70 and HSP90) [29]. In addition, adipocyte-enriched proteins such as adiponectin, fatty acid binding protein 4 (FABP4), and perilipin-1 have been detected in EV cargo derived from white adipocytes and circulating adipose EVs, although these markers are not exclusive and may reflect cellular origin rather than EV subtype specificity [28,30]. HSP70 and HSP90. In obesity and T2DM, exosomes from hypertrophic white adipocytes often acquire pro-inflammatory and insulin resistance-inducing properties [31].

#### 2.3.2. Brown Adipocytes

Brown adipocytes are multilocular thermogenic cells characterized by high mitochondrial content and expression of uncoupling protein-1 (UCP1) [32,33]. They release exosomes containing proteins and regulatory RNAs that promote energy expenditure, mitochondrial biogenesis, and metabolic homeostasis [34]. Similar to white adipocyte-derived EVs, brown Ad-EVs express canonical EV markers (CD9, CD63, CD81, ALIX, TSG101, HSP70, and HSP90), reflecting conserved EV biogenesis pathways. Importantly, several proteins have been proposed as putative brown adipose tissue-associated EV markers, including proton-coupled amino acid transporter-2 (PAT2), P2X purinoceptor-5 (P2RX5), and methylenetetrahydrofolate dehydrogenase (NADP^+^) 1-like (MTHFD1L), based on proteomic analyses of circulating EVs correlated with brown fat activity in humans and experimental models [35]. While these markers are not exclusively expressed on brown Ad-EVs, their enrichment has been associated with brown adipose tissue activation and thermogenic function. Although brown Ad-EXs are often regarded as metabolically protective under physiological conditions, emerging evidence suggests that in T2DM, their cargo and functional effects are altered. For example, brown Ad-EXs from diabetic mouse models impair aortic endothelial function through lowering intracellular calcium signaling and NO production to cause vascular endothelium-dependent relaxation dysfunction [36].

#### 2.3.3. Beige (Or Brite) Adipocytes

Beige adipocytes arise within white adipose depots under stimuli such as fibroblast growth factor 21 (FGF21), β-adrenergic agonists, cold exposure, or exercise. They share features of both white and brown adipocytes and express UCP1 upon activation [37]. Due to their close developmental and functional similarity to brown adipocytes, beige adipocytes are presumed to release extracellular vesicles with molecular and functional characteristics comparable to those of brown adipocyte-derived EVs. However, direct evidence defining beige adipocyte-derived EV surface markers remains limited, and no markers have been conclusively shown to distinguish beige Ad-EVs from brown Ad-EVs. Available studies suggest that EVs released from thermogenically active adipocytes carry bioactive cargo capable of reprogramming recipient tissues toward enhanced oxidative metabolism and improved metabolic homeostasis, thereby contributing to protection against obesity-associated metabolic dysfunction [34,38].

### 2.4. Adipocyte-Derived Exosomes in T2DM

**Figure 2:**
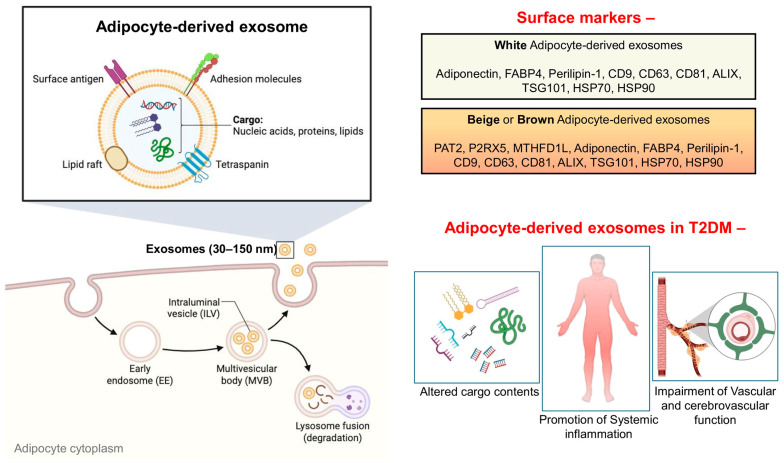
Biogenesis of adipocyte-derived exosomes and their role in T2DM. Adipocyte-derived exosomes (Ad-EXs) are formed through inward budding of the cytoplasm of the adipocyte cell and are released in the extracellular space via the exocytosis process. These Ad-EXs express different surface markers depending on their types (white, beige, or brown) with similar ultimate effects in T2DM to cause alteration in miRNA contents, promotion of systemic circulation, and disruption of vascular and cerebrovascular functions. Abbreviations—PAT2: proton-coupled amino acid transporter 2; P2RX5: P2X purinoceptor 5; MTHFD1L: methylenetetrahydrofolate dehydrogenase (NADP^+^) 1-like; FABP4: fatty acid binding protein 4; ALIX: ALG-2-interacting protein X; TSG101: tumor susceptibility gene 101; HSP70: heat shock protein 70; HSP90: heat shock protein 90. (Created in Biorender.com).

In T2DM, the metabolic and inflammatory state of adipose tissue is altered, leading to changes in the quantity and molecular cargo of Ad-EXs (Figure 2). Key features include altered cargo contents, promotion of systemic inflammation, and impairment of vascular and cerebrovascular function.

#### 2.4.1. Altered Cargo Contents

Alterations of cargo contents of Ad-EXs in T2DM are likely a contributing factor to metabolic dysregulation. The miRNA profile of healthy vs. T2DM patients is found to be very different from one another [39,40,41]. These miRNAs in circulation and in exosomal cargo regulate gene expression by blocking translation of specific messenger RNAs into proteins, and they are known to play an essential role in neurovascular injury and repair of various CNS disorders [42]. Insulin resistance (IR) is a pathogenic driver of T2DM and substantially influences both the quantity and molecular composition of circulating exosomes [43]. IR alters cellular metabolic signaling in key insulin-responsive tissues such as adipose, liver, and skeletal muscle, promoting chronic low-grade inflammation, dysregulated insulin signaling, and altered exosomal secretion profiles. For example, exosomes derived from adipose tissue under IR conditions are enriched with pro-inflammatory miRNAs and proteins that can propagate metabolic dysfunction by modulating macrophage polarization and inhibiting insulin signaling pathways in recipient cells [44]. Similarly, circulating exosomal miRNAs have been shown to differ in individuals with IR and T2DM, mediating inter-organ crosstalk that further exacerbates systemic IR and glucose intolerance [45,46]. These findings imply that the metabolic state of the donor cell, especially under conditions of impaired insulin action, not only increases exosome release but also reshapes exosomal cargo, thereby influencing pathophysiological processes relevant to T2DM development and complications. Ad-EXs derived from insulin-resistant or hypertrophic adipocytes show differential expression of miRNAs that regulate glucose metabolism, insulin signaling, and inflammatory pathways in recipient cells, including hepatocytes, pancreatic β-cells, skeletal cells, endothelial cells, and neurons. Lee et al. revealed a differential expression of 509 proteins, including 81 known adipokines, in adipocyte-derived extracellular vesicles of Otsuka Long-Evans Tokushima Fatty (OLETF) rats via nano-liquid chromatography with tandem mass spectrometric proteomic analyses [47]. The data showed 128 elevated proteins (such as aquaporin-7 and caveolin-1), alongside 72 reductions in proteins (such as catalase and adenylate kinase 2), suggesting shifts in lipid metabolism and oxidative stress response in target cells. Additionally, exosomes originating from visceral and subcutaneous adipose tissue of individuals with obesity and T2DM modulate the expression of cholesterol efflux-related genes (such as upregulating ABCG1 while downregulating LXRα and PPARG) in human monocyte-derived macrophages, thereby affecting reverse cholesterol transport and potentially influencing cardiovascular risk [48].

#### 2.4.2. Promotion of Systemic Inflammation

Pro-inflammatory cytokines and miRNAs transported via Ad-EXs can exacerbate chronic low-grade inflammation, characteristic of T2DM, contributing to vascular and metabolic complications [49]. In diabetic and obese conditions, hypertrophic adipocytes release exosomes enriched in pro-inflammatory cargo such as miR-34a, miR-155, and ceramides, which actively reprogram immune and metabolic cells. Exosomal miR-34a suppresses Krüppel-like factor 4 (KLF4) in macrophages, thereby inhibiting anti-inflammatory M2 polarization and promoting M1 polarization, leading to enhanced secretion of TNF-α, IL-6, and IL-1β [50]. Beyond immune modulation, Ad-EXs can influence endothelial cells by upregulating adhesion molecules such as VCAM-1 and ICAM-1, thereby fostering leukocyte recruitment and vascular inflammation. These exosomal signals extend to peripheral tissues, where they disrupt insulin signaling in the liver and skeletal muscle through PPARγ repression and JNK activation, leading to further systemic inflammatory conditions [23]. Collectively, these findings highlight that Ad-EXs act as endocrine-like messengers that bridge adipose tissue dysfunction with systemic inflammation, ultimately aggravating insulin resistance, vascular complications, and multi-organ injury in T2DM.

#### 2.4.3. Impairment of Vascular and Cerebrovascular Function

T2DM is widely known to cause vascular complications via endothelial dysfunction. Ad-EXs influence endothelial cell function, vascular permeability, and blood–brain barrier integrity, linking adipose tissue dysfunction to cerebrovascular complications commonly observed in T2DM [13]. Perivascular adipose tissue not only provides structural support to the blood vessels (hence vasculature) but also plays a dynamic role in the regulation of vascular homeostasis with its released adipokines (and EXs). Exosomal miRNAs from Ad-EXs play a key role in regulating the integrity of the vasculature and endothelial function throughout the body [51]. Tang et al. reported that exosomal miR-27b-3p secreted by visceral Ad-EXs activates the NF-κB pathway by downregulating PPARα in endothelial cells, both in vitro in human umbilical vein endothelial cells (HUVECs) and in vivo in ApoE mice, to promote inflammation and atherosclerosis [52]. miR-181b is studied for its positive involvement in the regulation of endothelial function via upregulation of endothelial nitric oxide synthase (eNOS) production and reduction of inflammation via suppression of NF-κB [53]. More research regarding Ad-EX-derived miR-181b and its involvement in T2DM vascular complications could generate new therapeutic avenues for the treatment of T2DM-associated cerebral and cerebrovascular complications. Blumensatt et al. reported that upregulated exosomal miR-143 from diabetic Ad-EXs can cause impairment of insulin-mediated phosphorylation of Akt and eNOS, along with reduction in the expression of the oxysterol-binding protein-related protein 8 (ORP8), leading to induction of proliferative and pro-atherogenic alterations in human vascular smooth muscle cells (hVSMC) [54]. Apart from the miRNAs from the Ad-EXs, other cargo contents also lead to a negative impact on the vasculature system. Wang et al. reported that insulin-resistant Ad-EXs in T2DM cause increased angiogenesis of vasa vasorum to promote plaque burden in atherosclerosis via sonic hedgehog (Shh) protein upregulation in HUVECs and murine aortic endothelial cells (MAECs) [55]. Overall, Ad-EXs play a role in T2DM-associated vascular and cerebrovascular complications. Elucidating the mechanistic pathways through which Ad-EXs modulate cerebrovascular function in T2DM holds significant potential to uncover novel therapeutic targets and foster the development of innovative treatment strategies. Table 1 summarizes key implications of adipocyte-derived exosomal miRNAs in endothelial dysfunction, vascular inflammation, pro-atherogenic remodeling, and metabolic function throughout the body, highlighting their relevance to T2DM-associated vascular and cerebrovascular complications.

## 3. Adipose Tissue–Brain Axis in T2DM: Role of Adipocyte-Derived Exosomes

**Figure 3:**
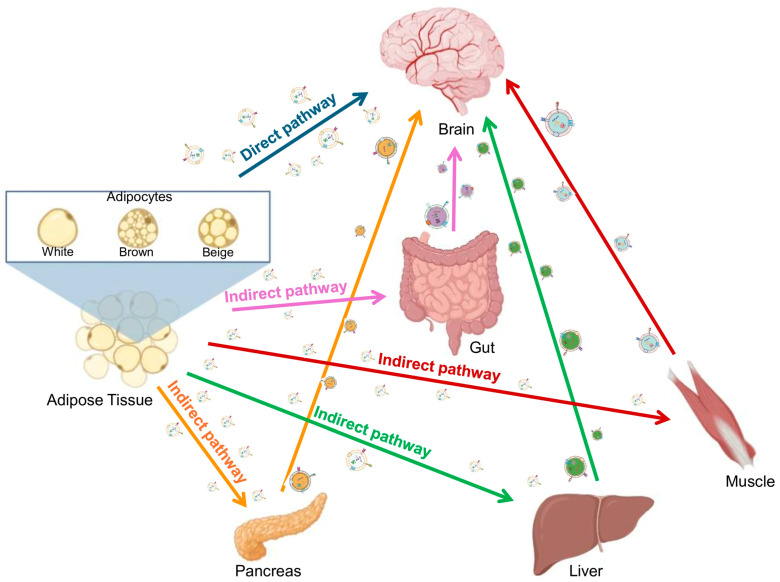
Adipocyte-derived exosomes in the adipose tissue–brain axis in T2DM. Direct pathway involves adipose tissue–brain communication via adipocyte-derived exosomes (Ad-EXs), and the indirect pathway involves adipose tissue communication with the pancreas, liver, muscle, and gut, which in turn can communicate with the brain via their derived exosomes (created in Biorender.com).

The adipose tissue–brain axis represents a key communication network through which adipose tissue can influence central nervous system (CNS) function, particularly under metabolic stress such as T2DM. Dysregulated adipose tissue in T2DM secretes altered bioactive factors, including adipokines, cytokines, and Ad-EXs, which can impact brain function both directly and indirectly (Figure 3).

### 3.1. Direct Pathway

The adipose tissue–brain axis is a complex, bidirectional communication network where adipose tissue and the brain can directly communicate with one another via various circulating factors, particularly Ad-EXs, which, after reaching the brain, interact with cellular components of the CNS [63,64]. In T2DM, circulating Ad-EXs are enriched with pro-inflammatory miRNAs and insulin signaling modulators, reflecting adipose tissue dysfunction. It is speculated that these Ad-EXs can cross the BBB via active and regulated processes, including endothelial transcytosis and receptor-mediated uptake, rather than passive diffusion. Studies on EV-BBB interactions indicate that vesicles bind to scavenger receptors (e.g., SR-A, CD36), heparan sulfate proteoglycans, and integrins expressed on brain microvascular endothelial cells, facilitating vesicle adhesion and internalization [65]. In addition, tetraspanins (CD9, CD63, CD81) present on EV membranes participate in vesicle docking and uptake specificity at the endothelial surface [66]. Following receptor engagement, EVs are internalized through clathrin-mediated endocytosis, caveolin-1-dependent transport, or macropinocytosis, enabling transcellular trafficking across endothelial cells while partially avoiding lysosomal degradation [67]. Direct screening approaches using Ad-EX uptake and tracking assays across BBB models would provide definitive evidence of Ad-EX cargo delivery to the brain parenchyma. Wang et al. reported that, in high-fat-diet-fed mice and T2DM patients, adipose tissue-derived EVs can cross the BBB and deliver miR-9-3p to the hippocampus, where they accumulate and trigger synaptic loss and cognitive decline by suppressing brain-derived neurotrophic factor (BDNF) expression. Notably, depleting miR-9-3p cargo content from adipose tissue-derived EVs attenuated these neurodegenerative and cognitive effects [16]. In T2DM, circulating EXs, including Ad-EXs, enriched with specific miRNAs or pro-inflammatory factors, impair endothelial barrier function by downregulating tight junction protein expression (ZO-1, occluding, claudin-5), thereby enhancing BBB permeability [68,69]. Importantly, as the adipose tissue–brain axis is bidirectional, accumulating evidence indicates that brain-derived exosomes also influence adipose tissue biology [70]. Neuronal and glial exosomes released from metabolically relevant brain regions, including the hypothalamus, can enter the circulation and modulate adipocyte insulin sensitivity, inflammatory signaling, lipolysis, and thermogenic programming [64,71]. These reciprocal exchanges are thought to cause a dynamic brain–adipose communication network that may contribute to systemic metabolic homeostasis and its dysregulation in obesity and T2DM. Brain-derived exosomes carry bioactive cargo such as miRNAs (e.g., miR-9, let-7 family, miR-21, miR-33), which can be delivered to distant peripheral tissues to alter gene regulatory programs [72]. However, while EV-mediated brain–periphery communication via miRNAs is increasingly recognized, direct mechanistic evidence linking brain-derived EV signaling to adipose dysfunction in T2DM remains limited and warrants further investigation. To summarize, Ad-EXs can directly cross the BBB to fuse with the neurovascular unit, including neurons, astrocytes, endothelial cells, macrophages, etc., and cause alteration in their functioning, especially in metabolic disorders like obesity and T2DM [73].

### 3.2. Indirect Pathway

The indirect pathway involves adipose tissue influencing the brain function through intermediary organs that are critical for systemic metabolic homeostasis [74]. Importantly, beyond acting as isolated signaling entities, Ad-EXs in T2DM are likely to exert systemic effects by reshaping the composition, abundance, and functional properties of the circulating EV pool. Given that adipose tissue is a major contributor to plasma EVs [23], pathological Ad-EX release in T2DM may influence EV biogenesis, cargo loading, and signaling behavior in secondary organs [75]. This indirect modulation can result in the release of secondary organ-derived exosomes with altered miRNA, protein, and lipid profiles, thereby amplifying metabolic stress signals that ultimately reach the brain. Thus, changes in global circulating EV dynamics, rather than isolated cargo alterations, may represent a critical mechanism by which adipose tissue dysfunction propagates systemic and neurovascular impairment in T2DM. *Effects* via *the pancreas*—The adipose tissue–pancreas axis, also known as the adipo-insular axis, is a bidirectional communicatory pathway signaling through adipokines, insulin, leptin, and recently explored exosomes [76]. Gesmundo et al. found that Ad-EXs from murine and human adipocytes affect pancreatic β-cell function, influencing insulin secretion depending on the physiological state of adipose tissue [77]. Dysfunctional β-cells lead to hyperglycemia and systemic insulin resistance, which indirectly affects brain glucose uptake and metabolism. Moreover, Ad-EXs may alter the biogenesis of pancreatic-EXs, which can ultimately communicate with the brain through the pancreas–brain axis, further amplifying metabolic stress signals. This should be researched further to generate therapeutic targets. *Effects* via *liver*—Evidence shows that Ad-EXs can target hepatocytes, altering lipid metabolism and promoting non-alcoholic fatty liver disease (NAFLD) [78]. Moreover, hepatic insulin resistance contributes to hyperglycemia and chronic inflammation, indirectly exacerbating cerebrovascular stress. Increasing attention has been given to the liver–brain axis in the context of gastrointestinal, pancreatic, and hepatic disorders [79]. Emerging evidence indicates that liver-derived exosomes contribute to functional and inflammatory alterations in the brain [80]. Although direct evidence linking Ad-EXs to the liver–brain axis remains limited, it is plausible that Ad-EX-induced hepatic dysfunction alters the release and composition of liver-derived EXs, thereby indirectly affecting brain homeostasis. This represents an important area for future investigation. *Effects* via *gut and microbiome*—The adipose tissue–gut axis is widely being studied in various metabolic diseases, particularly in relation to microbiome crosstalk [81]. In parallel, the gut–brain axis is increasingly being appreciated due to the role of microbiota and their derived exosomes in various CNS diseases [82]. Even when a direct link between Ad-EXs and altered gut microbial content has not yet been fully established, considering adipose tissue contributes to a substantial proportion of circulating EXs [83], the role of Ad-EXs in the adipose tissue–gut–brain axis is undeniable. Given emerging experimental evidence for cross-kingdom communication between eukaryotic EVs and microbial-derived vesicles [84,85], it is reasonable to propose that Ad-EXs may indirectly influence gut microbiota composition or function, thereby modulating gut-derived EV signaling to the brain. This adipose tissue–gut–brain axis represents a rapidly evolving and highly relevant area of metabolic–neurovascular research. *Effects* via *skeletal muscle*—The adipose tissue–skeletal muscle crosstalk is highly governed by myokines and adipokines and plays a central role in systemic insulin sensitivity [86]. The pathophysiological effects of Ad-EXs on skeletal muscle are being vastly studied through their carried proteins and miRNAs [87]. The skeletal muscle-derived EXs are known to contribute to the maintenance of brain homeostasis and neural plasticity [88]. Hence, dysfunctional skeletal muscle secondary to pathological Ad-EXs’ signaling may indirectly affect the brain functions, including cerebrovascular integrity. Collectively, these indirect pathways highlight how adipose tissue dysfunction in T2DM can influence the brain through multi-organ EV-mediated communication networks. Exploring these interconnected axes may uncover novel therapeutic targets for preventing or mitigating cerebrovascular complications of T2DM.

## 4. Mechanisms Linking Adipocyte-Derived Exosomes to Cerebrovascular Complications of T2DM

**Figure 4:**
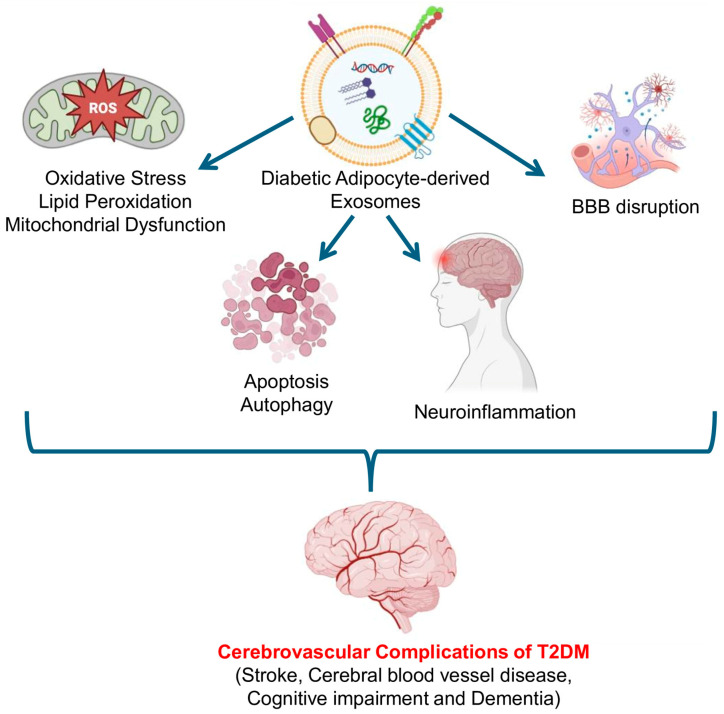
Mechanisms linking diabetic adipocyte-derived exosomes to cerebrovascular complications of T2DM. Adipocyte-derived exosomes are linked to cerebrovascular complications of T2DM through influencing mechanisms such as oxidative stress, lipid peroxidation, mitochondrial dysfunction, apoptosis, autophagy, neuroinflammation, and BBB disruption. (Created in Biorender.com).

Adipocyte-derived exosomes (Ad-EXs) act as endocrine messengers carrying lipids, proteins, and regulatory RNAs that influence distant cells of the brain [63]. In the setting of T2DM, adipocytes undergo metabolic stress, inflammation, and hypoxia, leading to altered exosome biogenesis and pathogenic cargo loading. Once released into circulation, these Ad-EXs can reach the neurovascular unit made up of various cells such as brain microvascular endothelial cells (BMECs), neurons, astrocytes, and pericytes—where they perturb homeostatic signaling. Mechanistically, diabetic Ad-EXs promote oxidative stress, lipid peroxidation, apoptosis, autophagy, mitochondrial dysfunction, neuroinflammation, and disruption of BBB integrity (Figure 4 and Table 2). They also deliver diabetogenic miRNAs that reprogram gene expression in the brain vasculature. Collectively, these pathways accelerate small-vessel disease, increase stroke susceptibility, and contribute to vascular cognitive impairment in diabetes.

### 4.1. Oxidative Stress, Lipid Peroxidation, and Mitochondrial Dysfunction

Oxidative stress is a central driver of cerebrovascular pathology in diabetes. Ad-EXs act as vectors that propagate pro-oxidant signals from adipose tissue to endothelial and neural cells. In T2DM, Ad-EXs enriched with ceramides and NOX4-related proteins activate NADPH oxidase in brain endothelial cells. This enhances the generation of superoxide and hydrogen peroxide, impairing nitric oxide bioavailability and vascular tone. Hypertrophic subcutaneous Ad-EXs contain NOX4, which causes severe oxidative stress in human trophoblast (HTR8/SVneo) cells, along with cellular senescence and suppressed proliferation and invasion functions [89]. Pillai et al. reported that Ad-EXs from LDL-treated oxidized adipocytes (murine 3T3-L1 and human adipose-derived MSC) can cause atherogenic phenotypes of macrophages (murine peritoneal macrophage and human monocyte), mitochondrial ROS production, and IL-6 cytokine secretion, which may ultimately lead to atherosclerosis and related diseases, including stroke [90]. In a study performed by He et al. on the effect of hyperglycemic Ad-EX on mouse retinal microvascular endothelial cells (mRMECs), it was found that LINC00968 from Ad-EXs causes mRMEC dysfunction by oxidative stress via the LINC00968/miR-361-5p/TRAF3 signaling pathway [91]. Another study reported by Wen et al. reported that hypertrophic adipocyte-derived exosomal miR-802-5p caused cardiac insulin resistance in neonatal rat ventricular myocytes through downregulating HSP60, which significantly increased the expression levels of C/EBP-homologous protein and enhanced oxidative stress, accompanied by the increases in the phosphorylation of JNK and IRS-1 Ser307 [61].

Lipid peroxidation represents a downstream effect of ROS imbalance, damaging cellular membranes and propagating vascular injury. Ceramides are considered a key lipotoxic component of adipose tissue, leading to global ROS production in T2DM conditions. Since Ad-EXs have a high ceramide composition as a cargo [92], ceramide-enriched Ad-EXs may accelerate lipid peroxidation in endothelial membranes by the accumulation of reactive aldehydes (4-hydroxyneonal; 4-HNE, malondialdehyde; MDA). Interestingly, a study by Zhang et al. has reported the protective effects of Ad-EXs from differentiated 3T3-L1 preadipocytes in mouse colorectal cancer (MC38) cells via downregulating MDA and lipid peroxidation through upregulation of microsomal triglyceride transfer protein (MTTP), which in turn reduces ferroptosis [93]. Moreover, the role of Ad-EXs in T2DM is still not clearly elucidated in regard to lipid peroxidation. However, it is speculated that diabetic Ad-EXs may cause lipid peroxidation to affect ion channels, receptors, and structural proteins, amplifying vascular dysfunction in atherosclerosis [103]. More research is needed to investigate the role of Ad-EX in T2DM in regard to lipid peroxidation because of its double-edged sword nature—pro-atherogenic nature via promotion of inflammation and anti-ferroptotic nature via suppression of lipid peroxidation.

Mitochondrial health is critical for cellular energy demands. Mitochondrial dysfunction is a well-established phenomenon in diabetes and is considered a key etiological factor contributing to diabetic complications, including cerebrovascular complications. Schpttl et al. have reported compromised mitochondrial capacity (lower respiration capacity and mitochondrial respiratory chain content as well as increased ROS generation) of VAT compared to SAT of C57BL/6N mice on a high-fat diet regimen [94]. On the Ad-EXs levels, Lee et al. reported that diabetic OLETF rats’ adipose-derived EXs carry various mitochondrial proteins such as mitochondrial fission protein 1 (FIS1), cytochrome oxidase IV-subunit I (cox-1), etc., as well as various lipids and nucleic acids, which can cause mitochondrial impairment of the recipient cells [47]. Ad-EXs miRNAs are also studied for their involvement in affecting mitochondrial bioenergetics. One of the recent studies by Gan et al. reported that diabetic Ad-EXs promote oxidative stress and mitochondria-mediated apoptosis of cardiomyocytes in ischemic heart injury via miR-130b-3p-induced suppression of PGC-1α expression in cardiomyocytes both in vitro and in vivo [95]. Interestingly, a study by Crewe et al. described a phenomenon termed ‘inter-organ mitohormesis’, in which mitochondria-associated components such as mitochondrial proteins (Translocase of the outer mitochondrial membrane 20; TOM20, Cytochrome c oxidase subunit IV; COX IV, ATP synthase subunit alpha; ATP5A), mitochondrial DNA, and mitochondrial fragments are transferred through energetically stressed Ad-EXs to cardiomyocytes for preconditioning of the heart against ischemia/reperfusion injury [104]. This study provided data regarding the transfer of damaged mitochondrial material via Ad-EXs in obese conditions from differentiated 3T3-L1 preadipocytes and adipocyte-specific mitochondrial reporter mice (adipo-FtMT mice) to the cardiomyocytes to trigger ROS, which functions as a mitohormetic signal rather than a cytotoxic insult. The ROS burst activates redox-sensitive stress-adaptive pathways, including NRF2-dependent anti-oxidant responses and upregulation of mitochondrial quality control and cytoprotective genes, thereby enhancing the cardiomyocyte’s capacity to withstand subsequent ischemia/reperfusion injury. Hence, Ad-EXs should be studied more for their involvement in mitochondrial signaling, which can contribute to altered mitochondrial dynamics and metabolic adaptation in T2DM.

### 4.2. Apoptosis and Autophagy

Programmed cell death of endothelial and neural cells is a hallmark of diabetic microvascular injury. In diabetes, Ad-EXs carry altered molecular cargos, which trigger apoptotic pathways in distant cells. As discussed earlier, exosomes from diabetic or high-glucose-treated adipocytes exacerbate apoptotic death in cardiomyocytes via mitochondrial dysfunction: a study showed that exosomal miR-130b-3p suppresses PGC-1α, leading to increased mitochondrial reactive oxygen species and activation of caspases and TUNEL-positive cell death in ischemia/reperfusion-injured hearts [95]. Similarly, Ad-EXs, via their ceramide and ROS-rich contents in T2DM, may promote mitochondrial cytochrome c release, further accelerating apoptosis of recipient cells. Moreover, as discussed earlier, Gesmundo et al. reported that Ad-EVs from obese and diabetic conditions in mice and humans cause detrimental effects on pancreatic β cells, shifting them toward death or dysfunction (increased apoptosis) compared to EVs from healthy adipocytes [77]. Thus, diabetic Ad-EXs act as mediators of apoptosis via mitochondrial damage in multiple target cell types and could be highly speculated to have similar effects in the neurovascular unit. Further research is needed to confirm this hypothesis.

Autophagy is a homeostatic process for cellular quality control, but under diabetic stress, exosomes from various origins can trigger maladaptive autophagy [105,106]. There are two main autophagy regulatory pathways—PI3K-Akt-mammalian rapamycin target protein (mTOR) and AMPK-mTOR. Although no studies to date have directly investigated how Ad-EXs regulate autophagy in the diabetic brain, emerging literature suggests a plausible intersection of these pathways. Under many pathological conditions, including T2DM, abnormal autophagy of various cells can cause the release of harmful exosomes from them [96]. Impaired autophagy of adipocytes in T2DM is well reported [105,107], and it can lead to the release of harmful Ad-EXs, which can trigger maladaptive autophagy in the CNS that harms the BBB. Uptake of diabetic Ad-EXs by endothelial cells may alter autophagic flux, leading to degradation of tight junction proteins (occludin, zo-1, claudin-5) [98]. This dysregulation could transform autophagy from a protective mechanism into a contributor to barrier dysfunction. More research should focus on finding the link between Ad-EXs and autophagy (especially the PI3K-Akt-mTOR or AMPK-mTOR pathways) in the neurovascular unit in the context of T2DM.

### 4.3. Neuroinflammation

Neuroinflammation links systemic metabolic dysfunction to progressive cognitive decline. Ad-EXs act as pro-inflammatory messengers within the neurovascular unit. Visceral fat–derived Ad-EXs carrying miR-27b-3p suppress PPARα, promoting endothelial adhesion molecule expression (VCAM-1, ICAM-1) to activate the NF-κB pathway, causing inflammation and atherogenesis [52]. These changes can also drive leukocyte infiltration and microglial activation, fostering chronic neuroinflammation. Song et al. reported that diabetic Ad-EXs carrying sonic hedgehog protein from differentiated 3T3-L1 cells cause pro-inflammatory M1 polarization of RAW 264.7 macrophages, leading to inflammation and insulin resistance via the Ptch and PI3K pathways [99]. These findings implicate Ad-EXs as mediators in the pathway from adipose dysfunction to neuroinflammation in T2DM.

### 4.4. BBB Integrity Disruption

BBB dysfunction is a defining feature of cerebrovascular complications in T2DM. Ad-EXs directly and indirectly compromise BBB integrity. Adipose-derived EVs can cross the BBB, altering communication between endothelial cells, pericytes, and astrocytes, thus weakening barrier selectivity. Studies of obesity, high-fat diet, and insulin resistance show that BBB integrity is compromised under those conditions, including reductions in tight junction proteins like ZO-1, Claudin-5, and Occludin, increased permeability, elevated matrix metalloproteinases (MMP2, MMP9), and increased systemic inflammation [100]. Wang et al. reported that Ad-EXs from 3T3-L1 cells promote murine 3LL Lewis lung cancer cell tumor invasion in vitro via transferring MMP3 as a cargo to elevate MMP9 activity [101]. Putting those together, a plausible pathway is that in diabetes/obesity, Ad-EXs with inflammatory cargo can either directly act on brain endothelial cells (altering tight junction expression to induce oxidative stress) or activate peripheral/intravascular inflammation that secondarily impairs BBB integrity. Elevated MMP activity induced by systemic inflammation could degrade the extracellular matrix and tight junctions, increasing leakage [102]. Based on the found pieces of evidence of the involvement of Ad-EX’s ability to cross the BBB, it is possible that the diabetic Ad-EXs would cause BBB disruption in the neurovascular unit to lead to cerebrovascular complications of T2DM. This paradigm should be tested to aid in the identification of a mechanistic pathway to unravel a therapeutic target for the treatment of cerebrovascular complications of T2DM.

## 5. Adipocyte-Derived Exosomes in a Specific Cerebrovascular Outcome

**Figure 5:**
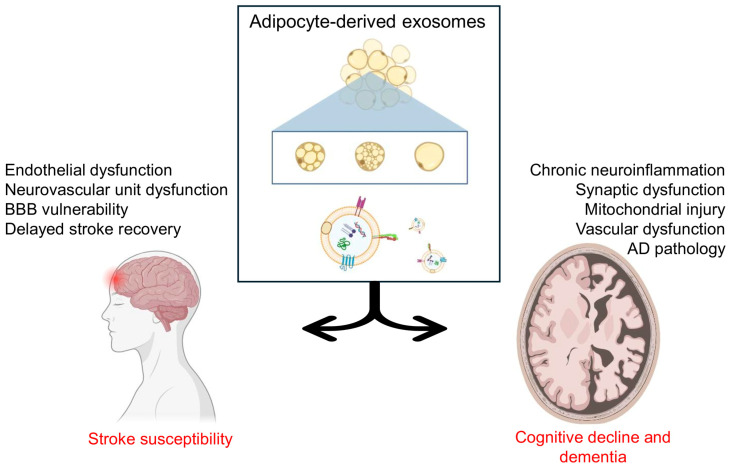
Adipocyte-derived exosomes in cerebrovascular outcomes. Adipocyte-derived exosomes (Ad-EXs) negatively influence two main outcomes of cerebrovascular functioning, i.e., stroke susceptibility and cognitive decline and dementia. Ad-EXs partake in inducing stroke susceptibility via endothelial and neurovascular unit dysfunction, BBB vulnerability, and delayed stroke recovery. Ad-EXs also lead to cognitive decline and dementia in T2DM by inducing chronic neuroinflammation, synaptic and vascular dysfunction, mitochondrial injury, and Alzheimer’s disease pathology. (Created in Biorender.com).

T2DM leads to various cerebrovascular complications due to its risk factors, such as hyperglycemia, hyperlipidemia, and hyperinsulinemia. These risk factors lead to vascular dysfunction, endothelial injury, formation of AGEs, and accelerated atherosclerosis in the cerebral circulation. As a result, individuals with T2DM have a markedly higher susceptibility to strokes, cerebral small vessel disease, and vascular cognitive impairment. While the mechanistic actions of Ad-EXs in T2DM converge on oxidative stress, inflammation, mitochondrial dysfunction, and BBB disruption, their downstream consequences manifest in distinct cerebrovascular outcomes. Two of the most clinically relevant are stroke, as the leading cause of mortality and disability in diabetes, and cognitive decline/dementia, as a major contributor to long-term morbidity (Figure 5).

### 5.1. Stroke Susceptibility

Individuals with T2DM exhibit a 2–4-fold increased risk of ischemic stroke and worse functional outcomes compared with non-diabetic populations [108,109,110]. T2DM is associated with impaired fibrinolysis, altered platelet function, and dysregulated coagulation pathways, further predisposing patients to both vascular rupture and poor hematoma resolution [111,112]. During an ischemic event, the diabetic brain is particularly vulnerable to injury due to increased BBB disruption, neuroinflammation, and impaired neurovascular repair mechanisms. Consequently, stroke outcomes in patients with T2DM are often worse, with higher rates of disability, recurrence, and mortality. Beyond conventional risk factors such as hypertension and dyslipidemia, Ad-EXs may provide a mechanistic link between metabolic dysfunction and heightened stroke susceptibility. Diabetic Ad-EXs carrying pro-inflammatory cytokines, miR-34a, and miR-27b-3p heighten endothelial oxidative stress, impair nitric oxide (NO) signaling, and upregulate adhesion molecules (VCAM-1, ICAM-1). This creates a pro-thrombotic and pro-inflammatory vascular environment, favoring plaque rupture and microvascular occlusion. As discussed earlier, Ad-EX-mediated disruption of tight junctions weakens the cerebral vasculature, making the BBB more prone to ischemic damage and hemorrhagic transformation during acute stroke events. Experimental evidence suggests that altered exosomal signaling in diabetes limits angiogenesis and repair processes via proteins that disrupt critical pathways like PI3K/Akt and VEGF, leading to larger infarcts and delayed recovery. Gan et al. reported that diabetic Ad-EXs via their cargo of miR-130b-3p and through downstream targets AMPKα1/α2, Ucp3, and Birc6 exacerbate myocardial ischemia/reperfusion injury by delayed recovery, larger infarct size, and greater apoptosis [113]. As the circulating EXs mostly originate from adipocytes, diabetic Ad-EXs likely play a crucial role in exhibiting the increased risk of strokes.

### 5.2. Cognitive Decline and Dementia

T2DM doubles the risk of developing dementia, with both vascular dementia and Alzheimer’s disease (AD) being accelerated by cerebrovascular injury. Ad-EXs have emerged as mediators of systemic-to-brain signaling that fuels neurodegeneration and cognitive decline. Ad-EXs carrying inflammatory miRNAs (e.g., miR-155, miR-27b-3p) activate microglia and astrocytes, sustaining a pro-inflammatory milieu that impairs synaptic plasticity. Batabyal et al. reported that miRNAs carried by Ad-EXs in patients with AD may downregulate cyclic AMP response element binding protein (CREB, a transcription factor for brain-derived neurotrophic factor; BDNF) signaling pathway involved in synaptic plasticity and memory formation [114]. Ad-EX delivery of miRNAs such as miR-34a could impair neuronal survival pathways (SIRT1, Bcl-2), contributing to dendritic spine loss and synaptic weakening, which is a known substrate for memory impairment [115]. Exosomal ceramides and stress-inducing RNAs compromise neuronal bioenergetics, leading to cumulative mitochondrial dysfunction that underlies cognitive decline. Ad-EX-driven BBB disruption and microvascular rarefaction promote chronic cerebral hypoperfusion and white matter lesions, characteristic of vascular cognitive impairment. A recent study by Yang et al. reported that serum exosomal microRNA-125a-5p, originating from adipose tissue, is upregulated in T2DM patients and is causally associated with an increased risk of Alzheimer’s disease (AD) and amnestic mild cognitive impairment (aMCI). Higher levels of this microRNA were linked to reduced hippocampal volume and poorer cognitive performance, with diagnostic accuracy for aMCI reaching an area under the curve (AUC) of 0.738 [116]. Diabetic exosomes may interact with amyloid and tau pathways, enhancing protein aggregation and accelerating Alzheimer-like neurodegeneration.

## 6. Clinical Implications

The growing recognition of Ad-EXs as critical mediators of metabolic and cerebrovascular dysfunction in T2DM opens new avenues for clinical translation. Their dual role as both disease biomarkers and potential therapeutic agents positions them at the forefront of precision medicine for T2DM-related cerebrovascular complications.

### 6.1. Adipocyte-Derived Exosomes as Biomarkers

Early detection of cerebrovascular complications in T2DM remains challenging, as conventional biomarkers (glucose, HbA1c, lipids) poorly reflect neurovascular health. Ad-EXs, accessible through blood or cerebrospinal fluid, provide a minimally invasive “liquid biopsy” that captures dynamic adipose tissue–brain communication [117]. The early clinical study by Burlacu et al. in ischemic stroke cohorts has identified circulating exosomal miRNAs such as miR-21, miR-29b, miR-125b-5p, miR-126, and miR-335 that are significantly associated with stroke severity and functional and cognitive recovery trajectories in patients, supporting their translational relevance as prognostic biomarkers [118]. In parallel, the cargo specificity of Ad-EXs makes them a unique and reliable biomarker, as exosomal miRNAs (e.g., miR-34a, miR-27b-3p, miR-155) and proteins (e.g., FABP4, ceramides) are selectively enriched in diabetic adipocytes and potentially Ad-EXs, correlating with vascular injury, BBB dysfunction, and inflammation. Levels of specific EV cargo linked to dysfunctional adipose tissue, including pro-inflammatory miRNAs and lipid mediators, have been shown in preclinical stroke models to correlate with infarct volume, endothelial injury, and post-ischemic neurological deficits. In parallel, early clinical studies in metabolic disease and ischemic stroke cohorts report associations between circulating exosomal miRNA profiles and adverse neurological and cognitive outcomes, supporting the potential predictive value of adipose tissue-associated EV cargo [119,120]. As discussed earlier, according to a recent study by Yang et al., serum exosomal miR-125a-5p derived from adipose tissue can serve as a causal biomarker for cognitive impairment in T2DM patients [116]. The advantage of Ad-EXs over free biomarkers is that, as compared to circulating proteins or RNAs, Ad-EX cargo is more stable due to vesicular protection, making them reliable longitudinal markers [121].

### 6.2. Adipocyte-Derived Exosomes as Therapeutic Agents

Beyond diagnostic potential, Ad-EXs are increasingly explored as therapeutic tools due to their natural ability to cross biological barriers, including the BBB, and deliver bioactive cargo to target cells [122], especially the brain [123]. Depending on the origin of Ad-EXs, their effects vary from bad to neutral to good. Zhou et al. reported that brown adipose tissue-EXs mitigate metabolic syndrome in HFD mice [34]. Adipose-derived stem cell (ADSC)-EXs are found to be protective against many diseases; for example, a recent study by Tang et al. reported that ADSC-EXs ameliorate traumatic brain injury via the NLRP3 pathway [124]. Based on this evidence of potential beneficial effects of Ad-EXs, engineering Ad-EXs to deliver protective miRNAs (e.g., miR-126, miR-132, miR-210) may promote endothelial repair, angiogenesis, and BBB stabilization to restore vascular integrity in diabetic stroke. To modulate inflammation, Ad-EXs enriched with anti-inflammatory molecules (e.g., miR-146a, IL-10 mRNA) could attenuate microglial activation and neuroinflammation, reducing vascular cognitive impairment. Delivery of exosomal anti-oxidants or mitochondrial-stabilizing RNAs through Ad-EXs could improve neuronal bioenergetics and reduce oxidative stress. Using the biocompatibility and ability to cross the BBB, Ad-EXs can be engineered to carry neuroprotective drugs, siRNAs, or CRISPR components to target cerebrovascular injury sites. Approaching Ad-EXs within the framework of personalized medicine holds considerable promise, as autologous exosome-based interventions may minimize immune rejection and enhance biocompatibility. Such patient-specific strategies could enable the development of tailored therapies for individuals with T2DM, offering precision in both metabolic regulation and therapeutic efficacy.

## 7. Knowledge Gaps

Although recent findings highlight the role of Ad-EXs in linking T2DM to cerebrovascular complications, our understanding remains incomplete. Several unresolved questions limit their translation into clinical practice. Addressing these gaps will be critical for advancing both basic science and therapeutic applications.

### 7.1. Mechanistic Gaps

It is not yet fully understood how adipocytes selectively package specific miRNAs, lipids, and proteins into exosomes under diabetic conditions, nor how this cargo changes across disease stages. Hence, cargo analysis of Ad-EXs will be instrumental in unraveling their vast potential in T2DM. Recently, many efforts have been made to contribute to shedding light on these Ad-EXs through omics studies, but they need the inclusion of T2DM-specific Ad-EX screening. Another gap is that the precise molecular cues that direct Ad-EXs to brain endothelial cells, neurons, or microglia are poorly defined, limiting efforts to harness or block these interactions. Whether Ad-EX-mediated signaling occurs early in T2DM progression or predominantly during advanced vascular injury remains unclear, raising a temporal dynamics question regarding Ad-EXs in T2DM.

### 7.2. Clinical and Translational Gaps

While several Ad-EX miRNAs and proteins are promising, large-scale, multi-ethnic, and longitudinal clinical studies are needed to validate their predictive value as a biomarker for stroke and dementia in T2DM patients. Lack of consensus on exosome isolation, quantification, and characterization (e.g., ultracentrifugation vs. immunocapture vs. microfluidics) hampers reproducibility across studies. Even the nomenclature variability, such as adipocyte-derived extracellular vesicles vs. adipocyte-derived exosomes, is contributing to confusion or unclear knowledge regarding Ad-EXs overall. It remains to be determined how Ad-EX biomarkers can complement neuroimaging, genomics, or traditional risk factors to improve risk stratification in clinical practice due to lack of incorporation using clinical tools.

### 7.3. Therapeutic Development Gaps

Efficient and reproducible loading of therapeutic cargo into Ad-EXs remains a significant technical challenge, with limited reports demonstrating robust methodologies, highlighting the need for innovative engineering strategies. Moreover, concerns regarding off-target effects, immunogenicity, and potential metabolic perturbations underscore the importance of rigorous preclinical evaluation to ensure therapeutic precision and safety. Industrial-scale production of clinical-grade exosomes with consistent quality remains a bottleneck. Regulatory frameworks for exosome-based therapeutics are still evolving, creating uncertainty for clinical translation. Addressing these challenges through advanced bioengineering approaches, scalable biomanufacturing platforms, and harmonized regulatory guidelines will be pivotal in harnessing Ad-EXs as safe and effective therapeutic agents.

## 8. Future Prospective

Despite growing evidence implicating the importance of Ad-EXs in the cerebrovascular complications of T2DM, significant knowledge gaps remain unresolved. Future studies should focus on delineating the precise cargo signatures of Ad-EXs from adipose tissue-derived EVs that drive endothelial dysfunction, neuroinflammation, and blood–brain barrier disruption. Multi-omics integration could be performed to combine exosomal transcriptomics, proteomics, and lipidomics of Ad-EXs, revealing comprehensive signatures of T2DM-related cerebrovascular risks. Advanced single-vesicle profiling and spatial transcriptomics could help uncover Ad-EX cell-type-specific effects in the brain. Longitudinal clinical studies are needed to validate Ad-EXs as reliable biomarkers for early detection of stroke risk, vascular cognitive impairment, and dementia in diabetic patients. Moreover, engineering patient-derived Ad-EXs as targeted delivery vehicles offers a promising therapeutic avenue for personalized medicine with high efficiency and low rejection incidences, though challenges related to vesicle heterogeneity, large-scale isolation, and safety must be addressed. A deeper mechanistic understanding and translational integration of Ad-EX research could ultimately transform the management of diabetes-associated neurovascular impairment.

## 9. Conclusions

Adipocyte-derived exosomes (Ad-EXs) have emerged as critical mediators linking metabolic dysfunction in T2DM to neurovascular impairment. Ad-EXs are different from adipose tissue-derived EXs (which are a mixture of EXs from various cells of adipose tissue), and they carry bioactive molecules such as microRNAs, proteins, and lipids, which can influence endothelial function, neuroinflammation, blood–brain barrier integrity, and neuronal survival, thereby contributing to stroke susceptibility, cognitive decline, and dementia in T2DM. Their dual role as both pathogenic drivers and potential therapeutic agents underscores the importance of understanding their mechanistic involvement. Further research into Ad-EX biology and their direct effects with mechanistic pathways on the neurovascular unit could pave the way for novel diagnostic biomarkers and exosome-based interventions to mitigate neurovascular complications in T2DM. Bridging knowledge gaps will require multidisciplinary collaboration across endocrinology, neurology, nanomedicine, and bioengineering. With continued advances, Ad-EX research holds the potential to revolutionize the prediction, prevention, and treatment of cerebrovascular complications in T2DM.

## Figures and Tables

**Table 1:** Effects of adipocyte-derived exosomal miRNAs on vascular and metabolic functions. Abbreviations—Ad-EXs: adipocyte-derived exosomes; EV: extracellular vesicle; PPARα: peroxisome proliferator-activated receptor alpha; NF-κB: nuclear factor kappa-light-chain-enhancer of activated B cells; HUVECs: human umbilical vein endothelial cells; ApoE^−^/^−^: apolipoprotein E knockout; Cacna1c: L-type Ca^2+^ channel subtype CaV1.2; eNOS: endothelial nitric oxide synthase; Akt: AKT Serine/Threonine Kinase; ORP8: oxysterol-binding protein-related protein 8; PI3K: phosphoinositide 3-kinase; IRS-1: Insulin Receptor Substrate 1; GLUT4: Glucose Transporter Type 4; Hsp60: heat shock protein 60.

miRNA	Ad-EXs Source	Target Cells	Molecular/Relevant Pathway	Effect	Experimental Model	Reference
miR-27b-3p	Visceral adipocyte-derived exosomes	Endothelial cells	PPARα downregulation; NF-κB activation	Promotes endothelial inflammation and atherosclerosis	HUVECs (in vitro); ApoE^−^/^−^ mice (in vivo)	[52]
miR-27a-5p	Visceral adipocyte-derived exosomes	Pancreatic β-cell	Inhibition of insulin secretion via Cacna1c	Elicits glucose intolerance by reducing β-cell insulin output	Mouse and human islets; in vitro and in vivo	[56]
miR-181b	Adipocyte-derived exosomes	Endothelial cells	eNOS upregulation; NF-κB suppression	Improves endothelial function and reduces vascular inflammation	Endothelial cell models	[57]
miR-143	Diabetic adipocyte-derived exosomes	Vascular smooth muscle cells (VSMCs)	Akt/eNOS signaling; ORP8 downregulation	Induces pro-atherogenic and proliferative vascular remodeling	Human VSMCs (in vitro)	[54]
miR-27a	Adipocyte-derived exosomes	Skeletal muscle	PPARγ downregulation	Promotes insulin resistance via impaired glucose uptake	Diet-induced obesity mice; C2C12 cells	[58]
miR-141-3p	Adipose exosomes (decreased in obesity)	Hepatocytes	PI3K/AKT modulation	Reduces insulin signaling and glucose metabolism	Ob/ob and HFD mouse adipose exosomes	[59]
miR-222	Gonadal white adipose tissue exosomes	liver and skeletal muscle tissues	IRS-1/GLUT4 modulation	Promotes insulin resistance in metabolic tissues	Obese mice	[60]
miR-34a	Adipocyte exosomes	adipose-resident macrophages	M2 polarization inhibition by repressing Klf4	Promotes adipose inflammation and systemic insulin resistance	Obese mouse adipocytes	[50]
miR-802-5p	Adipose EVs	Cardiac myocytes	Hsp60/metabolic signaling	Impairs insulin sensitivity in cardiac tissue	Rat model	[61]
miR-22-3p	Circulating adipose EVs	Skeletal muscle/metabolic tissues	AKT/IRS-1 signaling	Impairs insulin signaling	Mouse model	[62]

**Table 2:** Mechanisms, roles, and implications of adipocyte-derived exosomes in cerebrovascular complications of T2DM. Abbreviations—Ad-EXs: adipocyte-derived exosomes; NOX4: NADPH oxidase 4; IL6: Interleukin-6; ROS: reactive oxygen species; T2DM: type 2 diabetes mellitus; MDA: Malondialdehyde; 4HNE: 4-hydroxy-2-nonenal; MTTP: microsomal triglyceride transfer protein; FIS1: mitochondrial fission 1 protein; cox1: Cyclooxygenase-1; AMPK: AMP-activated protein kinase; PGC1α: peroxisome proliferator-activated receptor-γ coactivator 1-α; TNF-α: Tumor Necrosis Factor-alpha; PI3K: phosphoinositide 3-kinase; mTOR: mammalian target of rapamycin; NF-κB: nuclear factor kappa-light-chain-enhancer of activated B cells; NLRP3: NACHT, LRR, and PYD domains-containing protein 3; MMP: matrix metalloproteinase; ZO-1: Zonula Occludens-1.

Mechanism	Role of Adipocyte-Derived Exosomes (Ad-EXs)	Implications for Cerebrovascular Complications in T2DM	References
Oxidative Stress	Ad-EX miRNAs (e.g., miR-361-5p, miR-802-5p), LINC00968, NOX4, and IL6 downregulate anti-oxidant defense pathways, increasing ROS in T2DM	Promotes endothelial dysfunction, senescence, atherosclerosis, and stroke susceptibility	[61,89,90,91]
Lipid Peroxidation	Ad-EX ceramides, MDA, 4HNE, and MTTP induce lipid peroxidation in endothelial membranes in T2DM	Speculated induction of ROS and apoptosis of neurovascular unit	[92,93]
Mitochondrial Dysfunction	Ad-EXs transfer mitochondrial proteins (e.g., FIS1, cox1) and miRNAs that suppress mitophagy (e.g., via AMPK/PGC1α inhibition) in T2DM	Speculated reduced ATP production, excess ROS, heightened ischemic vulnerability in neurovascular unit	[47,94,95]
Apoptosis	Ad-EX’s pro-apoptotic cargo (TNF-α, miR-130b-3p) activate caspase cascades and inhibit survival pathways in T2DM	Speculated neurovascular unit apoptosis contributes to vascular cognitive impairment and ischemic injury	[77,95]
Autophagy Dysregulation	Ad-EX miRNAs and protein cargo may modulate autophagy regulators (PI3K, AMPK, mTOR) in T2DM	Speculated aberrant autophagy aggravates BBB damage, leading to stroke susceptibility and cognitive decline	[96,97,98]
Neuroinflammation	Ad-EX’s cargo TNF-α, and miR-27b-3p activate NF-κB and NLRP3 inflammasome in microglia/endothelium in T2DM	Sustained neuroinflammation drives BBB breakdown, white matter damage, and vascular cognitive impairment	[52,99]
BBB Disruption	Ad-EX cargo MMP (MMP2/3/9) impair tight junction proteins (occludin, claudin-5, ZO-1) in T2DM	Speculated BBB leakage facilitates infiltration of toxic mediators, worsening cerebrovascular injury	[100,101,102]

## Data Availability

No new data were created or analyzed in this study. Data sharing is not applicable to this article.

## References

[1] World Health Organization Diabetes. https://www.who.int/health-topics/diabetes.

[2] International Diabetes Federation (2025). IDF Diabetes Atlas.

[3] Cade W.T. (2008). Diabetes-Related Microvascular and Macrovascular Diseases in the Physical Therapy Setting. Phys. Ther..

[4] Hatzitolios A.I., Didangelos T.P., Zantidis A.T., Tziomalos K., Giannakoulas G.A., Karamitsos D.T. (2009). Diabetes Mellitus and Cerebrovascular Disease: Which Are the Actual Data?. J. Diabetes Its Complicat..

[5] Zhao M., Dong Y., Chen L., Shen H. (2024). Influencing Factors of Stroke in Patients with Type 2 Diabetes: A Systematic Review and Meta-Analysis. PLoS ONE.

[6] Mavridis A., Viktorisson A., Eliasson B., von Euler M., Sunnerhagen K.S. (2025). Risk of Ischemic and Hemorrhagic Stroke in Individuals with Type 1 and Type 2 Diabetes: A Nationwide Cohort Study in Sweden. Neurology.

[7] Isaman D.J.M., Herman W.H., Ye W. (2021). Prediction of Transient Ischemic Attack and Minor Stroke in People with Type 2 Diabetes Mellitus. J. Diabetes Its Complicat..

[8] Teng Z., Feng J., Liu R., Dong Y., Chen H., Xu J., Jiang X., Li R., Lv P. (2022). Cerebral Small Vessel Disease Is Associated with Mild Cognitive Impairment in Type 2 Diabetes Mellitus. Diabetes Metab. Syndr. Obes. Targets Ther..

[9] Lyu F., Wu D., Wei C., Wu A. (2020). Vascular Cognitive Impairment and Dementia in Type 2 Diabetes Mellitus: An Overview. Life Sci..

[10] Zhang B., Yang Y., Xiang L., Zhao Z., Ye R. (2019). Adipose-derived Exosomes: A Novel Adipokine in Obesity-associated Diabetes. J. Cell. Physiol..

[11] Rausch J., Horne K.E., Marquez L. (2025). The Effects of Adipose Tissue Dysregulation on Type 2 Diabetes Mellitus. Biomedicines.

[12] Yáñez-Mó M., Siljander P.R.-M., Andreu Z., Zavec A.B., Borràs F.E., Buzas E.I., Buzas K., Casal E., Cappello F., Carvalho J. (2015). Biological Properties of Extracellular Vesicles and Their Physiological Functions. J. Extracell. Vesicles.

[13] Connolly K.D., Rees D.A., James P.E. (2021). Role of Adipocyte-Derived Extracellular Vesicles in Vascular Inflammation. Free Radic. Biol. Med..

[14] Malaguarnera M., Cauli O., Cabrera-Pastor A. (2025). Obesity and Adipose-Derived Extracellular Vesicles: Implications for Metabolic Regulation and Disease. Biomolecules.

[15] Chen L., Amraee F., Sadegh-Nejadi S., Saberian M., Ghahari S.A., Miao X., Lisco G., Afrisham R. (2025). Molecular Mechanisms Linking Adipose Tissue-Derived Small Extracellular Vesicles/Exosomes to the Development or Amelioration of Obesity, Insulin Resistance, and Diabetes-Related Complications. Eur. J. Med. Res..

[16] Wang J., Li L., Zhang Z., Zhang X., Zhu Y., Zhang C., Bi Y. (2022). Extracellular Vesicles Mediate the Communication of Adipose Tissue with Brain and Promote Cognitive Impairment Associated with Insulin Resistance. Cell Metab..

[17] Welsh J.A., Goberdhan D.C.I., O’Driscoll L., Buzas E.I., Blenkiron C., Bussolati B., Cai H., Di Vizio D., Driedonks T.A.P., Erdbrügger U. (2024). Minimal Information for Studies of Extracellular Vesicles (MISEV2023): From Basic to Advanced Approaches. J. Extracell. Vesicles.

[18] Van Niel G., D’Angelo G., Raposo G. (2018). Shedding Light on the Cell Biology of Extracellular Vesicles. Nat. Rev. Mol. Cell Biol..

[19] Liu Z., Yin R., Tian J. (2025). Extracellular Vesicles: Mechanisms and Prospects in Type 2 Diabetes and Its Complications. Front. Endocrinol..

[20] Kwan H.Y., Chen M., Xu K., Chen B. (2021). The Impact of Obesity on Adipocyte-Derived Extracellular Vesicles. Cell. Mol. Life Sci..

[21] Connolly K.D., Guschina I.A., Yeung V., Clayton A., Draman M.S., Von Ruhland C., Ludgate M., James P.E., Rees D.A. (2015). Characterisation of Adipocyte-Derived Extracellular Vesicles Released Pre- and Post-Adipogenesis. J. Extracell. Vesicles.

[22] Durcin M., Fleury A., Taillebois E., Hilairet G., Krupova Z., Henry C., Truchet S., Trötzmüller M., Köfeler H., Mabilleau G. (2017). Characterisation of Adipocyte-Derived Extracellular Vesicle Subtypes Identifies Distinct Protein and Lipid Signatures for Large and Small Extracellular Vesicles. J. Extracell. Vesicles.

[23] Thomou T., Mori M.A., Dreyfuss J.M., Konishi M., Sakaguchi M., Wolfrum C., Rao T.N., Winnay J.N., Garcia-Martin R., Grinspoon S.K. (2017). Adipose-Derived Circulating miRNAs Regulate Gene Expression in Other Tissues. Nature.

[24] Sandoval-Bórquez A., Carrión P., Hernández M.P., Pérez J.A., Tapia-Castillo A., Vecchiola A., Fardella C.E., Carvajal C.A. (2024). Adipose Tissue Dysfunction and the Role of Adipocyte-Derived Extracellular Vesicles in Obesity and Metabolic Syndrome. J. Endocr. Soc..

[25] Chait A., Den Hartigh L.J. (2020). Adipose Tissue Distribution, Inflammation and Its Metabolic Consequences, Including Diabetes and Cardiovascular Disease. Front. Cardiovasc. Med..

[26] Fang L., Guo F., Zhou L., Stahl R., Grams J. (2015). The Cell Size and Distribution of Adipocytes from Subcutaneous and Visceral Fat Is Associated with Type 2 Diabetes Mellitus in Humans. Adipocyte.

[27] Hu D., Cong X., Gao B., Wu Y., Shen Q., Chen L. (2024). The Visceral Fat Area/Subcutaneous Fat Area Ratio Is Positively Associated with Carotid Atherosclerosis in Patients with Type 2 Diabetes Mellitus. Endocr. Connect..

[28] Zhou Z., Tao Y., Zhao H., Wang Q. (2021). Adipose Extracellular Vesicles: Messengers From and to Macrophages in Regulating Immunometabolic Homeostasis or Disorders. Front. Immunol..

[29] Le Lay S., Rome S., Loyer X., Nieto L. (2021). Adipocyte-Derived Extracellular Vesicles in Health and Diseases: Nano-Packages with Vast Biological Properties. FASEB BioAdvances.

[30] Eguchi A., Lazic M., Armando A.M., Phillips S.A., Katebian R., Maraka S., Quehenberger O., Sears D.D., Feldstein A.E. (2016). Circulating Adipocyte-Derived Extracellular Vesicles Are Novel Markers of Metabolic Stress. J. Mol. Med..

[31] Matilainen J., Berg V., Vaittinen M., Impola U., Mustonen A.-M., Männistö V., Malinen M., Luukkonen V., Rosso N., Turunen T. (2024). Increased Secretion of Adipocyte-Derived Extracellular Vesicles Is Associated with Adipose Tissue Inflammation and the Mobilization of Excess Lipid in Human Obesity. J. Transl. Med..

[32] Ricquier D. (2011). Uncoupling Protein 1 of Brown Adipocytes, the Only Uncoupler: A Historical Perspective. Front. Endocrinol..

[33] Townsend K., Tseng Y.-H. (2012). Brown Adipose Tissue: Recent Insights into Development, Metabolic Function and Therapeutic Potential. Adipocyte.

[34] Zhou X., Li Z., Qi M., Zhao P., Duan Y., Yang G., Yuan L. (2020). Brown Adipose Tissue-Derived Exosomes Mitigate the Metabolic Syndrome in High Fat Diet Mice. Theranostics.

[35] Leow M.K.-S., Rengaraj A., Narasimhan K., Verma S.K., Yaligar J., Thu G.L.T., Sun L., Goh H.J., Govindharajulu P., Sadananthan S.A. (2022). Activated Brown Adipose Tissue Releases Exosomes Containing Mitochondrial Methylene Tetrahydrofolate Dehydrogenase (NADP Dependent) 1-like Protein (MTHFD1L). Biosci. Rep..

[36] Ruan X., Zhao W. (2025). Brown Adipocyte-Derived Exosomes in Type 2 Diabetes Mellitus Impair Endothelial Function via Regulating Intracellular Calcium Cycle. Front. Cardiovasc. Med..

[37] Wu J., Boström P., Sparks L.M., Ye L., Choi J.H., Giang A.-H., Khandekar M., Virtanen K.A., Nuutila P., Schaart G. (2012). Beige Adipocytes Are a Distinct Type of Thermogenic Fat Cell in Mouse and Human. Cell.

[38] Chen Y., Pfeifer A. (2017). Brown Fat-Derived Exosomes: Small Vesicles with Big Impact. Cell Metab..

[39] Afsharmanesh M.R., Mohammadi Z., Mansourian A.R., Jafari S.M. (2023). A Review of Micro RNAs Changes in T2DM in Animals and Humans. J. Diabetes.

[40] Nunez Lopez Y.O., Garufi G., Pasarica M., Seyhan A.A. (2018). Elevated and Correlated Expressions of miR-24, miR-30d, miR-146a, and SFRP-4 in Human Abdominal Adipose Tissue Play a Role in Adiposity and Insulin Resistance. Int. J. Endocrinol..

[41] Al-Mahayni S., Ali M., Khan M., Jamsheer F., Moin A.S.M., Butler A.E. (2023). Glycemia-Induced miRNA Changes: A Review. Int. J. Mol. Sci..

[42] Sawant H., Sun B., Mcgrady E., Bihl J.C. (2024). Role of miRNAs in Neurovascular Injury and Repair. J. Cereb. Blood Flow Metab..

[43] Freeman D.W., Noren Hooten N., Eitan E., Green J., Mode N.A., Bodogai M., Zhang Y., Lehrmann E., Zonderman A.B., Biragyn A. (2018). Altered Extracellular Vesicle Concentration, Cargo, and Function in Diabetes. Diabetes.

[44] Lei L.-M., Lin X., Xu F., Shan S.-K., Guo B., Li F.-X.-Z., Zheng M.-H., Wang Y., Xu Q.-S., Yuan L.-Q. (2021). Exosomes and Obesity-Related Insulin Resistance. Front. Cell Dev. Biol..

[45] Lu G., Gao H., Dong Z., Jiang S., Hu R., Wang C. (2023). Change Profiles and Functional Targets of MicroRNAs in Type 2 Diabetes Mellitus Patients with Obesity. Diabetes Metab. J..

[46] Lu T., Zheng Y., Chen X., Lin Z., Liu C., Yuan C. (2024). The Role of Exosome Derived miRNAs in Inter-Cell Crosstalk among Insulin-Related Organs in Type 2 Diabetes Mellitus. J. Physiol. Biochem..

[47] Lee J.-E., Moon P.-G., Lee I.-K., Baek M.-C. (2015). Proteomic Analysis of Extracellular Vesicles Released by Adipocytes of Otsuka Long-Evans Tokushima Fatty (OLETF) Rats. Protein J..

[48] Dracheva K.V., Pobozheva I.A., Anisimova K.A., Panteleeva A.A., Garaeva L.A., Balandov S.G., Hamid Z.M., Vasilevsky D.I., Pchelina S.N., Miroshnikova V.V. (2024). Extracellular Vesicles Secreted by Adipose Tissue during Obesity and Type 2 Diabetes Mellitus Influence Reverse Cholesterol Transport-Related Gene Expression in Human Macrophages. Int. J. Mol. Sci..

[49] Lintsen D., Broux B. (2026). Effects and Mechanisms of Adipose Tissue-Derived Extracellular Vesicles in Vascular Inflammation and Dysfunction. Neural Regen. Res..

[50] Pan Y., Hui X., Hoo R.L.C., Ye D., Chan C.Y.C., Feng T., Wang Y., Lam K.S.L., Xu A. (2019). Adipocyte-Secreted Exosomal microRNA-34a Inhibits M2 Macrophage Polarization to Promote Obesity-Induced Adipose Inflammation. J. Clin. Investig..

[51] Chen H.-H., Li H.-F., Tseng T.-L., Lin H. (2023). Perivascular Adipose Tissue and Adipocyte-Derived Exosomal miRNAs Maintain Vascular Homeostasis. Heliyon.

[52] Tang Y., Yang L.-J., Liu H., Song Y.-J., Yang Q.-Q., Liu Y., Qian S.-W., Tang Q.-Q. (2023). Exosomal miR-27b-3p Secreted by Visceral Adipocytes Contributes to Endothelial Inflammation and Atherogenesis. Cell Rep..

[53] Sun X., Lin J., Zhang Y., Kang S., Belkin N., Wara A.K., Icli B., Hamburg N.M., Li D., Feinberg M.W. (2016). MicroRNA-181b Improves Glucose Homeostasis and Insulin Sensitivity by Regulating Endothelial Function in White Adipose Tissue. Circ. Res..

[54] Blumensatt M., Wronkowitz N., Wiza C., Cramer A., Mueller H., Rabelink M.J., Hoeben R.C., Eckel J., Sell H., Ouwens D.M. (2014). Adipocyte-Derived Factors Impair Insulin Signaling in Differentiated Human Vascular Smooth Muscle Cells via the Upregulation of miR-143. Biochim. Biophys. Acta BBA-Mol. Basis Dis..

[55] Wang F., Chen F.-F., Shang Y.-Y., Li Y., Wang Z.-H., Han L., Li Y.-H., Zhang L., Ti Y., Zhang W. (2018). Insulin Resistance Adipocyte-Derived Exosomes Aggravate Atherosclerosis by Increasing Vasa Vasorum Angiogenesis in Diabetic ApoE-/- Mice. Int. J. Cardiol..

[56] Zhang Y., Qian B., Yang Y., Niu F., Lin C., Yuan H., Wang J., Wu T., Shao Y., Shao S. (2024). Visceral Adipocyte-Derived Extracellular Vesicle miR-27a-5p Elicits Glucose Intolerance by Inhibiting Pancreatic β-Cell Insulin Secretion. Diabetes.

[57] Wang S., Shi M., Zhou J., Wang W., Zhang Y., Li Y. (2022). Circulating Exosomal miR-181b-5p Promoted Cell Senescence and Inhibited Angiogenesis to Impair Diabetic Foot Ulcer via the Nuclear Factor Erythroid 2-Related Factor 2/Heme Oxygenase-1 Pathway. Front. Cardiovasc. Med..

[58] Yu Y., Du H., Wei S., Feng L., Li J., Yao F., Zhang M., Hatch G.M., Chen L. (2018). Adipocyte-Derived Exosomal MiR-27a Induces Insulin Resistance in Skeletal Muscle Through Repression of PPARγ. Theranostics.

[59] Dang S.-Y., Leng Y., Wang Z.-X., Xiao X., Zhang X., Wen T., Gong H.-Z., Hong A., Ma Y. (2019). Exosomal Transfer of Obesity Adipose Tissue for Decreased miR-141-3p Mediate Insulin Resistance of Hepatocytes. Int. J. Biol. Sci..

[60] Li D., Song H., Shuo L., Wang L., Xie P., Li W., Liu J., Tong Y., Zhang C.-Y., Jiang X. (2020). Gonadal White Adipose Tissue-Derived Exosomal MiR-222 Promotes Obesity-Associated Insulin Resistance. Aging.

[61] Wen Z., Li J., Fu Y., Zheng Y., Ma M., Wang C. (2020). Hypertrophic Adipocyte-Derived Exosomal miR-802-5p Contributes to Insulin Resistance in Cardiac Myocytes Through Targeting HSP60. Obesity.

[62] Zhang H., Zhang X., Wang S., Zheng L., Guo H., Ren Y., Qiao B., Wu J., Zhao D., Xu L. (2023). Adipocyte-Derived Exosomal miR-22-3p Modulated by Circadian Rhythm Disruption Regulates Insulin Sensitivity in Skeletal Muscle Cells. J. Biol. Chem..

[63] Yerrapragada S.M., Bihl J.C. (2022). Role of Exosomes in Mediating the Cross-Talk Between Adipose Tissue and the Brain. Neuromolecular Med..

[64] Díaz-Castro F., Morselli E., Claret M. (2024). Interplay between the Brain and Adipose Tissue: A Metabolic Conversation. EMBO Rep..

[65] Joshi B.S., Zuhorn I.S. (2021). Heparan Sulfate Proteoglycan-Mediated Dynamin-Dependent Transport of Neural Stem Cell Exosomes in an in Vitro Blood-Brain Barrier Model. Eur. J. Neurosci..

[66] Abdelsalam M., Ahmed M., Osaid Z., Hamoudi R., Harati R. (2023). Insights into Exosome Transport through the Blood-Brain Barrier and the Potential Therapeutical Applications in Brain Diseases. Pharmaceuticals.

[67] Ramos-Zaldívar H.M., Polakovicova I., Salas-Huenuleo E., Corvalán A.H., Kogan M.J., Yefi C.P., Andia M.E. (2022). Extracellular Vesicles through the Blood-Brain Barrier: A Review. Fluids Barriers CNS.

[68] Yoo D.Y., Yim H.S., Jung H.Y., Nam S.M., Kim J.W., Choi J.H., Seong J.K., Yoon Y.S., Kim D.W., Hwang I.K. (2016). Chronic Type 2 Diabetes Reduces the Integrity of the Blood-Brain Barrier by Reducing Tight Junction Proteins in the Hippocampus. J. Vet. Med. Sci..

[69] Osaid Z., Haider M., Hamoudi R., Harati R. (2023). Exosomes Interactions with the Blood-Brain Barrier: Implications for Cerebral Disorders and Therapeutics. Int. J. Mol. Sci..

[70] Zhou W., Zhao L., Mao Z., Wang Z., Zhang Z., Li M. (2023). Bidirectional Communication Between the Brain and Other Organs: The Role of Extracellular Vesicles. Cell. Mol. Neurobiol..

[71] Huo L., Du X., Li X., Liu S., Xu Y. (2021). The Emerging Role of Neural Cell-Derived Exosomes in Intercellular Communication in Health and Neurodegenerative Diseases. Front. Neurosci..

[72] D’Amico G., Carista A., Manna O.M., Paladino L., Picone D., Sarullo S., Sausa M., Cappello F., Vitale A.M., Caruso Bavisotto C. (2024). Brain-Periphery Axes: The Potential Role of Extracellular Vesicles-Delivered miRNAs. Biology.

[73] Isaac R., Reis F.C.G., Ying W., Olefsky J.M. (2021). Exosomes as Mediators of Intercellular Crosstalk in Metabolism. Cell Metab..

[74] Crewe C., Scherer P.E. (2022). Intercellular and Interorgan Crosstalk through Adipocyte Extracellular Vesicles. Rev. Endocr. Metab. Disord..

[75] Diez-Roda P., Perez-Navarro E., Garcia-Martin R. (2024). Adipose Tissue as a Major Launch Spot for Circulating Extracellular Vesicle-Carried MicroRNAs Coordinating Tissue and Systemic Metabolism. Int. J. Mol. Sci..

[76] Kim J., Oh C.-M., Kim H. (2023). The Interplay of Adipokines and Pancreatic Beta Cells in Metabolic Regulation and Diabetes. Biomedicines.

[77] Gesmundo I., Pardini B., Gargantini E., Gamba G., Birolo G., Fanciulli A., Banfi D., Congiusta N., Favaro E., Deregibus M.C. (2021). Adipocyte-Derived Extracellular Vesicles Regulate Survival and Function of Pancreatic β Cells. JCI Insight.

[78] Duwaerts C.C., Maher J.J. (2019). Macronutrients and the Adipose-Liver Axis in Obesity and Fatty Liver. Cell. Mol. Gastroenterol. Hepatol..

[79] Matsubara Y., Kiyohara H., Teratani T., Mikami Y., Kanai T. (2022). Organ and Brain Crosstalk: The Liver-Brain Axis in Gastrointestinal, Liver, and Pancreatic Diseases. Neuropharmacology.

[80] Han W., Zhang H., Feng L., Dang R., Wang J., Cui C., Jiang P. (2023). The Emerging Role of Exosomes in Communication between the Periphery and the Central Nervous System. MedComm.

[81] Borozan S., Fernandez C.J., Samee A., Pappachan J.M. (2025). Gut-Adipose Tissue Axis and Metabolic Health. Curr. Issues Mol. Biol..

[82] Sun B., Sawant H., Borthakur A., Bihl J.C. (2023). Emerging Therapeutic Role of Gut Microbial Extracellular Vesicles in Neurological Disorders. Front. Neurosci..

[83] Yi C.-X., Tschöp M.H. (2012). Brain-Gut-Adipose-Tissue Communication Pathways at a Glance. Dis. Model. Mech..

[84] Luo R., Chang Y., Liang H., Zhang W., Song Y., Li G., Yang C. (2023). Interactions between Extracellular Vesicles and Microbiome in Human Diseases: New Therapeutic Opportunities. iMeta.

[85] Huang J., Yu Y., Feng Z., Yin Y., Liu Y., Liu X., Yu R. (2025). Cross-Kingdom Dialogue of Microbial Messengers: Multi-Target Regulatory Mechanisms and Therapeutic Strategies of Gut Microbiota-Derived Extracellular Vesicles in Metabolic Diseases. Int. J. Nanomed..

[86] Li F., Li Y., Duan Y., Hu C.-A.A., Tang Y., Yin Y. (2017). Myokines and Adipokines: Involvement in the Crosstalk between Skeletal Muscle and Adipose Tissue. Cytokine Growth Factor Rev..

[87] Yue B., Wang H., Cai X., Wang J., Chai Z., Peng W., Shu S., Fu C., Zhong J. (2022). Adipose-Secreted Exosomes and Their Pathophysiologic Effects on Skeletal Muscle. Int. J. Mol. Sci..

[88] Luo J., Pu Q., Wu X. (2024). Recent Advances of Exosomes Derived from Skeletal Muscle and Crosstalk with Other Tissues. Int. J. Mol. Sci..

[89] Tao Y., Chen W., Xu H., Xu J., Yang H., Luo X., Chen M., He J., Bai Y., Qi H. (2023). Adipocyte-Derived Exosomal NOX4-Mediated Oxidative Damage Induces Premature Placental Senescence in Obese Pregnancy. Int. J. Nanomed..

[90] Pillai S.S., Pereira D.G., Zhang J., Huang W., Beg M.A., Knaack D.A., de Souza Goncalves B., Sahoo D., Silverstein R.L., Shapiro J.I. (2023). Contribution of Adipocyte Na/K-ATPase A1/CD36 Signaling Induced Exosome Secretion in Response to Oxidized LDL. Front. Cardiovasc. Med..

[91] He W., Lin A., Wang C. (2023). Adipocyte-Derived Exosomal LINC00968 Promotes Mouse Retina Microvascular Endothelial Cell Dysfunction in a High-Glucose Environment by Modulating the miR-361-5p/TRAF3 Axis. Horm. Metab. Res. Horm. Stoffwechselforschung Horm. Metab..

[92] Horbay R., Hamraghani A., Ermini L., Holcik S., Beug S.T., Yeganeh B. (2022). Role of Ceramides and Lysosomes in Extracellular Vesicle Biogenesis, Cargo Sorting and Release. Int. J. Mol. Sci..

[93] Zhang Q., Deng T., Zhang H., Zuo D., Zhu Q., Bai M., Liu R., Ning T., Zhang L., Yu Z. (2022). Adipocyte-Derived Exosomal MTTP Suppresses Ferroptosis and Promotes Chemoresistance in Colorectal Cancer. Adv. Sci..

[94] Schöttl T., Kappler L., Braun K., Fromme T., Klingenspor M. (2015). Limited Mitochondrial Capacity of Visceral versus Subcutaneous White Adipocytes in Male C57BL/6N Mice. Endocrinology.

[95] Gan L., Zhao J., Yao P., Christopher T.A., Lopez B., Lau W.B., Koch W., Gao E., Ma X., Wang Y. (2025). Adipocyte-Derived Small Extracellular Vesicles Exacerbate Diabetic Ischemic Heart Injury by Promoting Oxidative Stress and Mitochondrial-Mediated Cardiomyocyte Apoptosis. Redox Biol..

[96] Xing H., Tan J., Miao Y., Lv Y., Zhang Q. (2021). Crosstalk between Exosomes and Autophagy: A Review of Molecular Mechanisms and Therapies. J. Cell. Mol. Med..

[97] Jakubek P., Pakula B., Rossmeisl M., Pinton P., Rimessi A., Wieckowski M.R. (2024). Autophagy Alterations in Obesity, Type 2 Diabetes, and Metabolic Dysfunction-Associated Steatotic Liver Disease: The Evidence from Human Studies. Intern. Emerg. Med..

[98] Kim K.-A., Kim D., Kim J.-H., Shin Y.-J., Kim E.-S., Akram M., Kim E.-H., Majid A., Baek S.-H., Bae O.-N. (2020). Autophagy-Mediated Occludin Degradation Contributes to Blood–Brain Barrier Disruption during Ischemia in bEnd.3 Brain Endothelial Cells and Rat Ischemic Stroke Models. Fluids Barriers CNS.

[99] Song M., Han L., Chen F., Wang D., Wang F., Zhang L., Wang Z., Zhong M., Tang M., Zhang W. (2018). Adipocyte-Derived Exosomes Carrying Sonic Hedgehog Mediate M1 Macrophage Polarization-Induced Insulin Resistance via Ptch and PI3K Pathways. Cell. Physiol. Biochem..

[100] Feng Z., Fang C., Ma Y., Chang J. (2024). Obesity-Induced Blood-Brain Barrier Dysfunction: Phenotypes and Mechanisms. J. Neuroinflamm..

[101] Wang J., Wu Y., Guo J., Fei X., Yu L., Ma S. (2017). Adipocyte-Derived Exosomes Promote Lung Cancer Metastasis by Increasing MMP9 Activity via Transferring MMP3 to Lung Cancer Cells. Oncotarget.

[102] Alexander J.S., Elrod J.W. (2002). Extracellular Matrix, Junctional Integrity and Matrix Metalloproteinase Interactions in Endothelial Permeability Regulation. J. Anat..

[103] Hennig B., Chow C.K. (1988). Lipid Peroxidation and Endothelial Cell Injury: Implications in Atherosclerosis. Free Radic. Biol. Med..

[104] Crewe C., Funcke J.-B., Li S., Joffin N., Gliniak C.M., Ghaben A.L., An Y.A., Sadek H.A., Gordillo R., Akgul Y. (2021). Extracellular Vesicle-Based Interorgan Transport of Mitochondria from Energetically Stressed Adipocytes. Cell Metab..

[105] Ferhat M., Funai K., Boudina S. (2019). Autophagy in Adipose Tissue Physiology and Pathophysiology. Antioxid. Redox Signal..

[106] Liu W., Xiao W., Zeng Q., Zhou Y., Yang J., Ouyang W. (2025). The Exosome-PI3K/Akt-Autophagy Axis in Diabetic Vascular Complications: Mechanisms and Implications. Diabet. Med..

[107] Butoyi C., Iqbal M.A., Boateng I.D. (2025). Latest Trends on Interplay of Autophagy, Adipose Tissue, and Gut Microbiota in Obesity-Related Metabolic Disorders. Hum. Nutr. Metab..

[108] Olofindayo J., Peng H., Liu Y., Li H., Zhang M., Wang A., Zhang Y. (2015). The Interactive Effect of Diabetes and Central Obesity on Stroke: A Prospective Cohort Study of Inner Mongolians. BMC Neurol..

[109] Sarwar N., Gao P., Seshasai S.R.K., Gobin R., Kaptoge S., Di Angelantonio E., Ingelsson E., Lawlor D.A., Selvin E., Emerging Risk Factors Collaboration (2010). Diabetes Mellitus, Fasting Blood Glucose Concentration, and Risk of Vascular Disease: A Collaborative Meta-Analysis of 102 Prospective Studies. Lancet.

[110] Jerkins T.W., Bell D.S.H. (2025). Stroke in the Patient with Type 2 Diabetes. Endocr. Pract..

[111] Kaur R., Kaur M., Singh J. (2018). Endothelial Dysfunction and Platelet Hyperactivity in Type 2 Diabetes Mellitus: Molecular Insights and Therapeutic Strategies. Cardiovasc. Diabetol..

[112] Bryk-Wiązania A.H., Undas A. (2021). Hypofibrinolysis in Type 2 Diabetes and Its Clinical Implications: From Mechanisms to Pharmacological Modulation. Cardiovasc. Diabetol..

[113] Gan L., Xie D., Liu J., Bond Lau W., Christopher T.A., Lopez B., Zhang L., Gao E., Koch W., Ma X.-L. (2020). Small Extracellular Microvesicles Mediated Pathological Communications Between Dysfunctional Adipocytes and Cardiomyocytes as a Novel Mechanism Exacerbating Ischemia/Reperfusion Injury in Diabetic Mice. Circulation.

[114] Batabyal R.A., Bansal A., Cechinel L.R., Authelet K., Goldberg M., Nadler E., Keene C.D., Jayadev S., Domoto-Reilly K., Li G. (2023). Adipocyte-Derived Small Extracellular Vesicles from Patients with Alzheimer Disease Carry miRNAs Predicted to Target the CREB Signaling Pathway in Neurons. Int. J. Mol. Sci..

[115] Kao Y.-C., Wang I.-F., Tsai K.-J. (2018). miRNA-34c Overexpression Causes Dendritic Loss and Memory Decline. Int. J. Mol. Sci..

[116] Yang S., Yuan Y., Zhang B., Wu T., Yu C., Li F., Zhu W., Zhai B., Zhang W., Wang J. (2025). Identification of Adipose Tissue-Derived Exosomal microRNA as a Novel Causal Biomarker for Cognitive Impairment in Type 2 Diabetes Mellitus: Triangulating Evidence from Mendelian Randomization and Multicentre Population Studies. Diabetes Obes. Metab..

[117] Li J., Song J., Jia L., Wang M., Ji X., Meng R., Zhou D. (2024). Exosomes in Central Nervous System Diseases: A Comprehensive Review of Emerging Research and Clinical Frontiers. Biomolecules.

[118] Burlacu C.-C., Ciobanu D., Badulescu A.-V., Chelaru V.-F., Mitre A.-O., Capitanescu B., Hermann D.M., Popa-Wagner A. (2022). Circulating MicroRNAs and Extracellular Vesicle-Derived MicroRNAs as Predictors of Functional Recovery in Ischemic Stroke Patients: A Systematic Review and Meta-Analysis. Int. J. Mol. Sci..

[119] Paneru B.D., Hill D.A. (2023). The Role of Extracellular Vesicle-Derived miRNAs in Adipose Tissue Function and Metabolic Health. Immunometabolism Cobham.

[120] Xie Y., Deng T., Xie L., Xie Y., Ma J., Zhong D., Huang X., Li Y. (2024). Effects of Extracellular Vesicles for Ischemic Stroke: A Meta-analysis of Preclinical Studies. Exp. Ther. Med..

[121] Le Lay S., Scherer P.E. (2025). Exploring Adipose Tissue-Derived Extracellular Vesicles in Inter-Organ Crosstalk: Implications for Metabolic Regulation and Adipose Tissue Function. Cell Rep..

[122] Mehdizadeh S., Mamaghani M., Hassanikia S., Pilehvar Y., Ertas Y.N. (2025). Exosome-Powered Neuropharmaceutics: Unlocking the Blood-Brain Barrier for next-Gen Therapies. J. Nanobiotechnol..

[123] Williams A., Branscome H., Kashanchi F., Batrakova E.V. (2025). Targeting of Extracellular Vesicle-Based Therapeutics to the Brain. Cells.

[124] Tang L., Xu Y., Wang L., Pan J. (2023). Adipose-Derived Stem Cell Exosomes Ameliorate Traumatic Brain Injury through the NLRP3 Signaling Pathway. Neuroreport.

